# Analysis of gastric microbiota and *Helicobacter pylori* infection in gastroesophageal reflux disease

**DOI:** 10.1186/s13099-022-00510-3

**Published:** 2022-09-13

**Authors:** Titong Sugihartono, Kartika Afrida Fauzia, Muhammad Miftahussurur, Langgeng Agung Waskito, Purwo Sri Rejeki, Reny I’tishom, Ricky Indra Alfaray, Dalla Doohan, Rizki Amalia, Camilia Metadea Aji Savitri, Yudith Annisa Ayu Rezkitha, Junko Akada, Takashi Matsumoto, Yoshio Yamaoka

**Affiliations:** 1 Division of Gastroenterology-Hepatology, Department of Internal Medicine, Faculty of Medicine-Dr. Soetomo Teaching Hospital, Universitas Airlangga, Jalan Mayjend Prof. Dr. Moestopo No. 6-8, Surabaya, 60286 Indonesia; 2 Department of Environmental and Preventive Medicine, Faculty of Medicine, Oita University, 1-1 Idaigaoka, Hasama-machi, Yufu, Oita 879-5593 Japan; 3 Department of Public Health and Preventive Medicine, Faculty of Medicine, Universitas Airlangga, Surabaya, 60131 Indonesia; 4 Helicobacter Pylori and Microbiota Study Group, Institute of Tropical Disease, Universitas Airlangga, Surabaya, 60115 Indonesia; 5 Department of Physiology and Medical Biochemistry, Faculty of Medicine, Universitas Airlangga, Surabaya, 60131 Indonesia; 6 Department of Internal Medicine, Faculty of Medicine, Universitas Airlangga, Surabaya, 60131 Indonesia; 7 Department of Anatomy, Histology and Pharmacology Faculty of Medicine, Universitas Airlangga, Surabaya, 60131 Indonesia; 8 Faculty of Medicine, University of Muhammadiyah Surabaya, Surabaya, 60113 Indonesia; 9 Department of Medical Biology, Faculty of Medicine, Universitas Airlangga, Surabaya, 60131 Indonesia; 10 Department of Medicine, Gastroenterology and Hepatology Section, Baylor College of Medicine, Houston, TX 77030 USA

**Keywords:** GERD, ERD, NERD, Microbiome, Helicobacter pylori, Infectious disease

## Abstract

**Background:**

We evaluated the microbiota in the stomach of Gastroesophageal Reflux Disease (GERD) patients. We compared Erosive Reflux Disease (ERD) to gastritis and Non-erosive Reflux Disease (NERD) subjects by 16S rRNA approach on gastric biopsy specimens. A total of 197 subjects were included consisting of gastritis (68; 34.52%), ERD (55; 27.92%), and NERD (74; 37.56%). After quality filtering, 187 samples were included for OTU analysis using Qiime2.

**Results:**

We observed a significant difference in alpha diversity (Shannon and Simpson indexes were *P* = 0.0016 and *P* = 0.017, respectively). A significant decrease in alpha diversity index was observed in NERD with *Helicobacter pylori (H. pylori)-*positive subjects than in gastritis (Simpson index *P* = 0.022; Shannon index *P* = 0.029), indicating a significant influence of *H. pylori* on the diversity in the stomach despite the diseases. In *H. pylori*-negative samples, alpha diversity measurement by the abundance coverage estimates (ACE) and Fisher Test revealed that ERD had significantly lower richness than gastritis and NERD groups (*P* = 0.00012 and *P* = 0.00043, respectively). *Anaerobacillus *sp. could only be found in ERD patients by LEFse analysis.

**Conclusions:**

The presence of ERD could alter microbiome diversity. A negative correlation between *H. pylori* and ERD is shown in this microbiome study but not in NERD.

**Supplementary Information:**

The online version contains supplementary material available at 10.1186/s13099-022-00510-3.

## Introduction

Among gastrointestinal diseases, gastroesophageal reflux disease (GERD) is the most prevalent disease in primary care facilities and often remains unsolved even after referral to secondary healthcare facilities. The reflux symptom was predominant and caused high morbidity, high medication cost and further disturbed patient’s productivity and quality of life [1, 2]. In GERD, gastric juice is frequently refluxed into the lower esophagus, predisposing to the erosion and transition of squamous epithelial mucosa [3]. Based on the present of erosion, GERD generally can be classified as Erosive Reflux Disease (ERD) and Non-erosive Reflux Disease (NERD) [4]. Together, GERD pathophysiology is mainly due to the impaired Lower Esophageal Sphincter function, other factors have been associated with GERD severity, such as impaired esophageal clearance and delayed gastric emptying [5]. In addition, obesity, high fat ingestion, alcohol-drinking habit, and smoking were reported to cause more severe acid reflux [6].

The microbiome is maintained in a particular composition in each part of the normal gastrointestinal tract. For example, transient Gram-positive bacteria predominate in the esophagus [7], while a more stable microorganism occupied the extremely low acidity of the gastric environment. The balance created by this commensalism affects the metabolism of microorganisms in the stomach environment and is significantly associated with diseases and phenotype [7]. A failure in maintaining microbiome composition will create a condition called dysbiosis. A previous study reported that gastric microorganisms produce a peptide that modulates ghrelin secretion. Ghrelin regulates the enterochromaffin cells that produce gastric juice, thus indirectly affecting the acidity level in the stomach [8]. Moreover, certain bacteria could catalyze gas that causes bloating and increase stomach pressure [9]. During GERD, the shifting of abundant microbiota to Gram-negative bacteria was already reported, but the study only evaluated changes in the esophagus [7, 10]. In comparison, microbiota in gastric antrum affecting gastric acidity was often overlooked.

In discussing gastrointestinal tract disease causes, *Helicobacter pylori* (*H. pylori)* is inseparable from gastric microbiota. It extensively occupies gastric mucosa and inhibits the growth of other bacteria. This remarkable shift in the gastric microbiota was associated with gastric cancer development [11]. However, *H. pylori* and GERD’s relationship are in continuous controversy. A recent meta-analysis showed an inverse correlation between the two, as the risk of GERD was increased after *H. pylori* eradication [12]. Indonesia was reported to have a low prevalence of *H. pylori* infection, while GERD prevalence was relatively high [13]. Conversely, in another study, *H. pylori* did not significantly associate with the GERD development [14]. These phenomena warrant further investigation from a microbiota perspective.

Recently, the development of next-generation sequencing technology facilitates and accelerates the investigation of microbiota. 16S rRNA approach using gastric biopsy specimens was used to evaluate stomach microbiota composition between GERD (either ERD or NERD) and gastritis patients. The role of *H. pylori* infection was also assessed. Further investigation that focused on non-*H. pylori* aspect was also conducted by excluding *H. pylori* from further analysis dataset. Since *H. pylori* could significantly affect the dataset, the excluding of this bacteria could give more clear explanation on which other bacteria that might have role on GERD. This gastric microbiota study could give insight into disease mechanisms and a stepping stone for developing both preventive and curative measures.

## Results

### Characteristics of the subjects

Subjects included in the study were classified into three groups: 68 gastritis, 55 ERD, and 74 NERD. The average age among groups was not significantly different (*P* > 0.05). According to diagnosis by histology and culture, *H. pylori* infection rate among groups was lowest in ERD (3/57, 5.2%) (Table 1). Alcohol drinking and smoking habits showed a significant association with diseases. The proportion of alcohol drinking and smoking is the highest in ERD subjects (44.9% and 43.0%, respectively).

**Table 1:** Subjects’ demographic data

Parameter		Gastritis (%)	GERD (%)	P-value^a^	NERD (%)	P-value^b^
Age	Median; IQR	51; 21.5	47; 20	0.53	44; 17	0.04*
Gender	Male	33 (48.5)	34 (61.8)	0.39	46 (62.2)	0.39
	Female	35 (51.5)	21 (21.4)		28 (37.8)	
*H. pylori* status	Positive	18 (26.4)	3 (5.5)	0.0092	17 (23.0)	0.70
	Negative	50 (73.5)	52 (32.7)		57 (77.0)	
Alcohol**	Yes	20 (30.3)	44 (80.0)	< 0.001	34 (46.0)	0.082
	No	46 (69.7)	11 (20.0)		40 (54.0)	
Smoking**	Yes	23 (34.8)	46 (83.6)	< 0.001	38 (51.4)	0.061
	No	43 (65.2)	9 (16.4)		36 (66.7)	

^a^P-value used the pairwise Fisher test association between GERD and Gastritis

^b^P-value used the pairwise Fisher test association between NERD and Gastritis

^*^Pairwise Wilcox test was used to compare the age between groups

^**^In this dataset, there were 2 missing value that was already adjusted

### Gastric microbiota diversity among gastritis, NERD, and ERD

The 16S rRNA sequencing yielded the average reads of 21,137 with minimum reads of 84 and maximum reads of 246,822. After quality filtering, analyzed data were set at a minimum of 500 reads in each sample and normalized by square root. Hence, 187 samples were included for further analysis. In this analysis, all samples in the dataset were included regardless of *H. pylori* infection status. In this dataset, 3113 taxa were detected and assigned to 23 phyla. The relative abundance of each phylum in each sample across groups is depicted (see Additional file 1: Fig. S2).

**Fig. 1:**
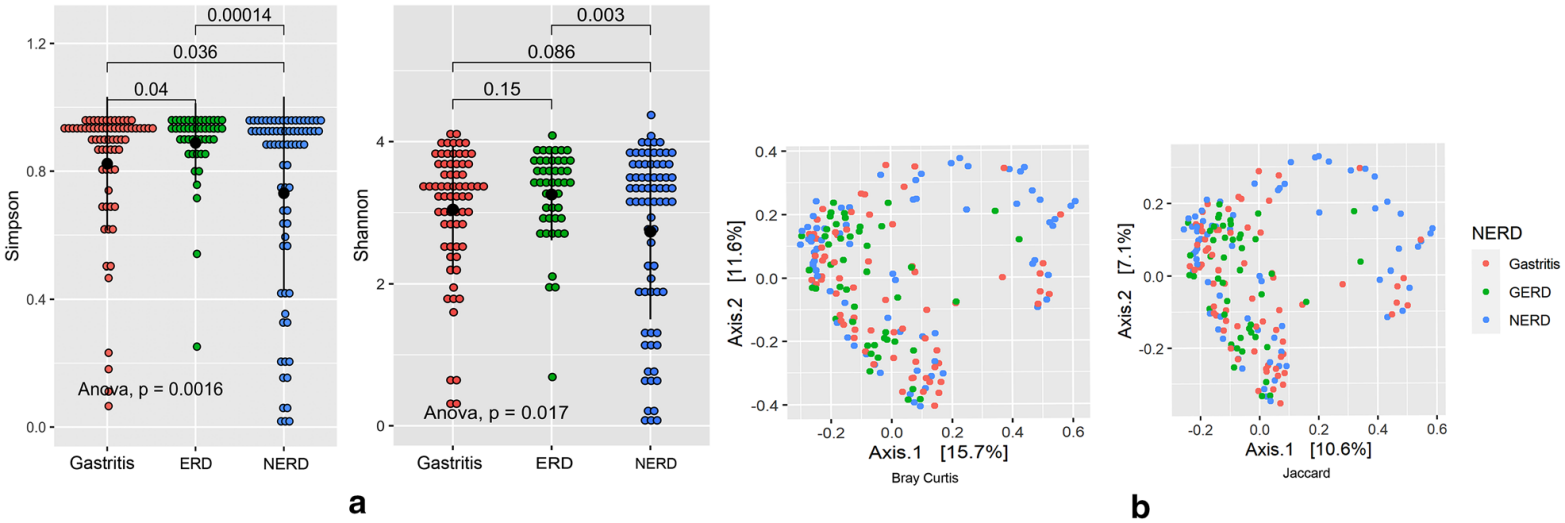
Alpha and beta diversity parameter of the dataset among gastritis, ERD, and NERD; **a**. Alpha diversity by Simpson and Shannon diversity shifted among diseases group; **b** Beta diversity by Bray–Curtis (left) and Jaccard (right) distance of all samples in the dataset

We observed a significant difference in alpha diversity between the Shannon index (P = 0.0016) and Simpson index (*P* = 0.017). The Simpson index of the ERD group was significantly higher than gastritis (*P* = 0.04) and NERD (*P* = 0.00014) (Fig. 1a). A similar pattern was also observed in the Shannon index, in which the diversity of the ERD group is significantly higher than to NERD (*P* = 0.003).

The beta diversity measured by Jaccard and Bray–Curtis also showed a significant shift by PERMANOVA (*P* < 0.001) due to the significant distance of specific samples from the remaining (Fig. 1b).

Alpha diversity was also compared among the severity of ERD according to the Los Angeles classification, LA-A, LA-B and LA-C. There were no LA-D found among the ERD in this dataset. A significantly higher Simpson index was observed in LA-C in comparison to LA-A (P = 0.036) (Additional file 1: Fig. S7).

### Diversity between H. pylori-positive and -negative

**Fig. 2:**
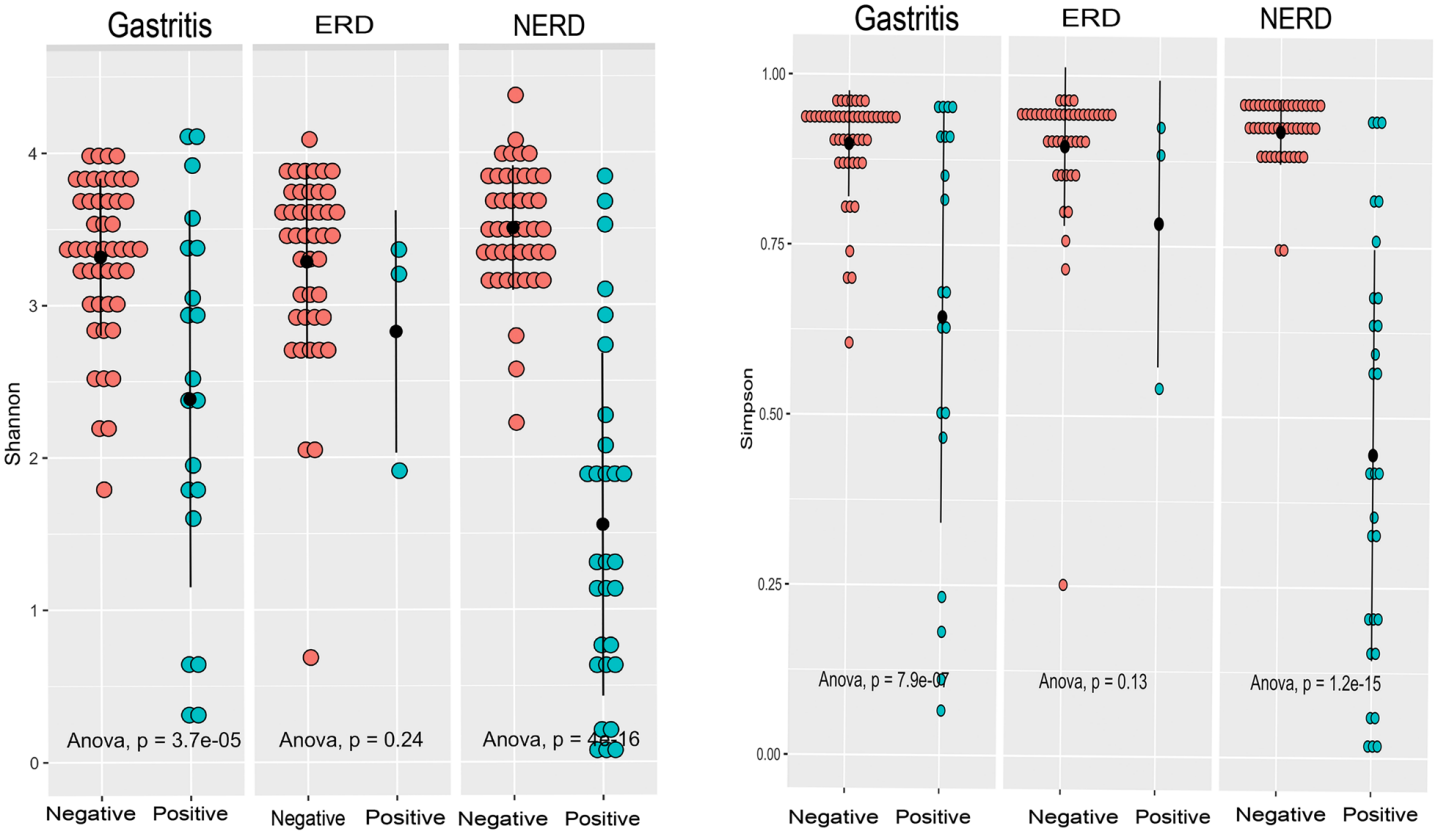
Alpha diversity in the presence and absence of *H. pylori. “*Negative” indicates a group of *H. pylori-*negative and the “Positive” showed *H. pylori* infection status, while the dot and line represented mean and standard error

To assess whether *H. pylori* affects the diversity, a separated analysis among *H. pylori*-positive (n = 135) and *H. pylori*-negative (n = 52) was performed. In this analysis, *H. pylori* was defined by ‘histology’ and ‘culture’ with microbial colonization termed as ‘an abundance of more than 2%’, as previously described [15]. Therefore, the proportion of *H. pylori*-positive in each group will increase, compared to the original criteria using only histology and culture. In the NERD group, the proportion of *H. pylori-*positive was 35.6% (26/73), while in the gastritis group was 32.4% (22/68), and the ERD group was only 8.7% (4/46). Despite the diseases, the diversity score was generally lower in *H. pylori*-positive groups than in *H. pylori*-negative groups (Fig. 2).

A distinct pattern was observed between *H. pylori-*negative and positive groups (Fig. 2). The Shannon index was significantly lower with *H. pylori*-negative in gastritis and NERD (*P* < 0.001 and *P* < 0.001, respectively). A significant decrease in the Simpson index was also concordant with the Shannon index. *H. pylori* appeared to give less impact on the alpha diversity in the ERD group (*P* = 0.24 for Shannon and *P* = 0.13 for Simpson indexes).

The beta diversity analysis by Unifrac distance, Bray–Curtis, and Jaccard indexes also showed that samples positioned in the furthest distance consisted of *H. pylori-*positive samples (See Additional File 1: Fig. S3). These results indicate influences of *H*. *pylori* on the diversity in the gastric environment. Furthermore, NERD samples showed the furthest distance of PC1, which indicates a substantial dissimilarity from the remaining groups.

### Microbiota diversity between ERD and NERD patients

Due to the significant influence of *H. pylori* presence in microbiome composition, 52 samples with confirmed *H. pylori* infection by histology, culture, and metagenomics-presence with an abundance of more than 2% were excluded for further analysis. After exclusion, the total taxa observed was 2778.

**Fig. 3:**
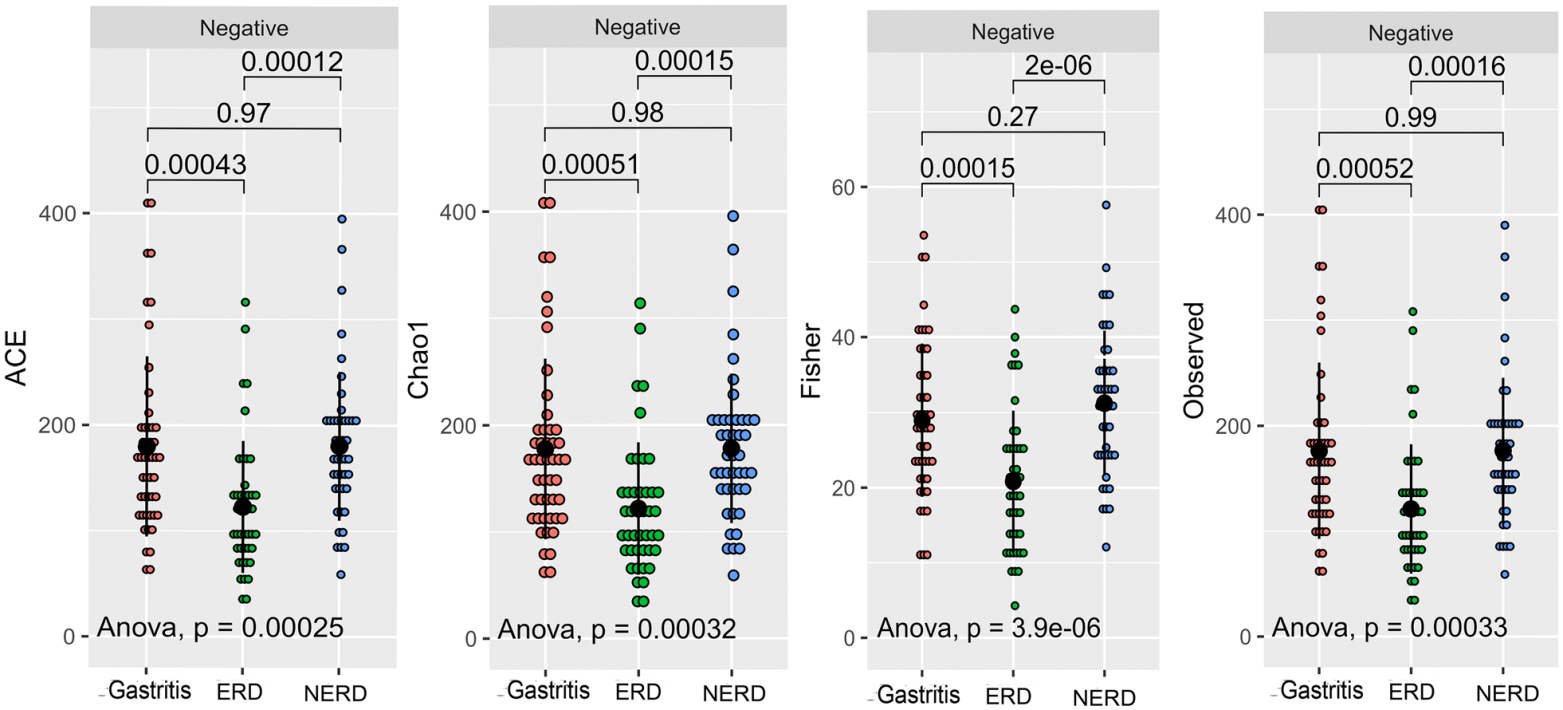
The alpha diversity measurement by ACE, Chao1, Fisher and Observed Richness index among *H. pylori-*negative patients. The black dot represents the average value and the bar shows the standard deviation

In *H. pylori-*negative samples, alpha diversity measurement by Shannon and Simpson indexes was not significantly altered between groups (Fig. 3). Hence, another diversity measurement accommodating taxa with low abundance was performed. The Abundance Coverage Estimates (ACE) revealed that ERD was significantly lower than the gastritis and NERD groups (*P* = 0.00012 and *P* = 0.00043, respectively). Similar results were also observed in the Chao1 index.

**Fig. 4:**
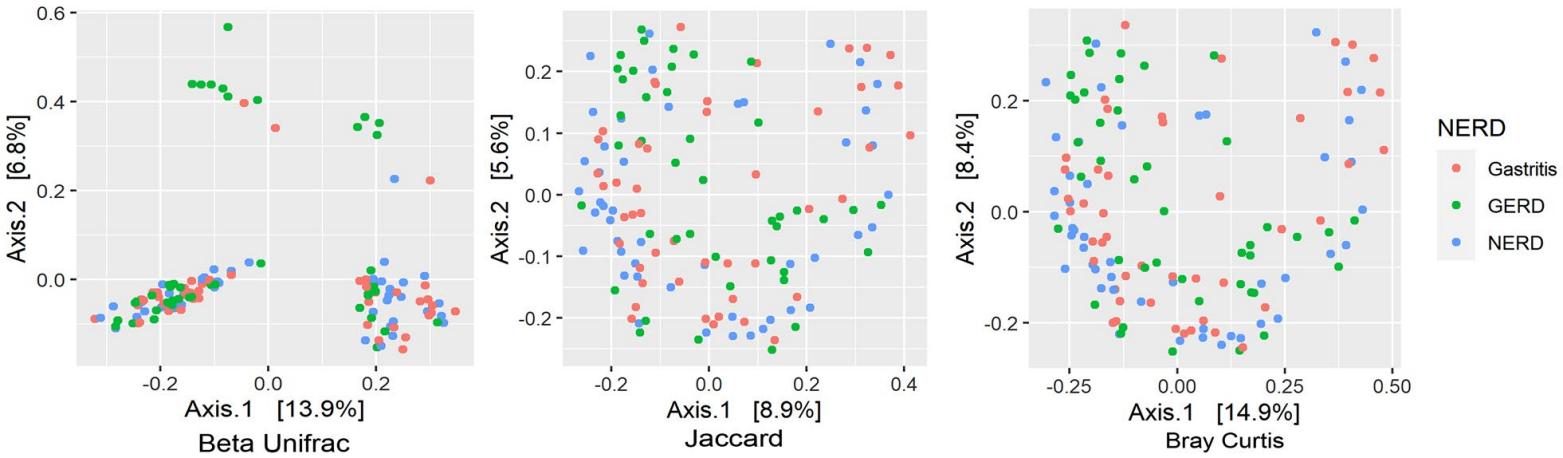
The beta diversity measurement (from left to right) Unifrac, Bray–Curtis, and Jaccard between gastritis, GERD, and NERD group after excluding the samples with *H. pylori*

Further analysis of the beta diversity using Unifrac, Bray–Curtis, and Jaccard distance among the *H. pylori-*negative group was concordant with the alpha diversity measurement (Fig. 4). The cluster was hardly separated in Bray–Curtis and Jaccard plot (*P* = 0.093 and *P* = 0.075). The Unifrac distance plot revealed a cluster of ERD group separated from the others, concordant with P < 0.001 by PERMANOVA analysis. ANCOM-BC analysis were also performed to find the taxa that significantly profound in ERD; such as *Treponema sp, Olsenella uli, Clostridium *sensu*-stricto, and Micrococcaceae.*

A comparison of relative abundance between groups showed *Firmicutes* as the highest abundance phylum (see Additional file 1: Fig. S4)*.* The most abundant class among *Firmicutes* is *Bacilli* which were predominant in all disease groups. There was no significant proportion shift when analyzing on order and family level, as *Lactobacillales* Order and *Streptococeae* Family predominated all groups. However, a significant increase in *Lactobacillus* proportion was observed only in the NERD group.

Linear discriminant analysis at the genus level was performed among taxa with an abundance of at least 2% in the population. Several taxa that characterize each disease group was observed with the LDA score cutoff of 4.0 (see Additional file 1: Fig. S5). In ERD, the highest LDA score was Gram-positive bacteria *Anaerobacillus *sp. and an unclassified genus from *Rhizobiaceae.* This *Anaerobacillus* was only found in the ERD group. Meanwhile, in the NERD group, the discriminatory taxa were *Rothia sp.* and *Alloprevotella *sp. (see Additional file 1: Fig. S5)*.*

## Discussion

Our study was conducted to understand the microbiome environment between GERD (either ERD or NERD) and gastritis. GERD might not be a life-threatening disease, but it can severely impair patients’ quality of life [16]. Our results showed that the microbiota diversity in gastric antrum between gastritis, ERD, and NERD subjects was significantly different. Moreover, we observed a substantial influence of *H. pylori* on microbiome diversity*.* After excluding samples containing *H. pylori,* the alpha diversity analysis results revealed that ERD patients had significantly lower microbial richness. This result is in concordance with a previous study of stomach microbiota, showing a relatively low diversity compared to other groups [17]. Mechanical and chemical changes during reflux, such as high acidity, delayed gastric emptying, increased stomach pressure due to gas production, and LES weakening was suspected of causing microbiome shift [18]. The excessive growth of other species in gastric mucosa could also be an alternate mechanism of dysbiosis [19].

We attempted to evaluate the diversity and bacteria other than *H. pylori* which can dominate the stomach environment. Our results showed that microbiota composition among ERD, NERD and gastritis groups did not diverge from the normal stomach. In ERD and gastritis group, the predominant phyla were *Firmicutes*, followed by *Proteobacteriota* and *Bacteroidota*. These phyla were known as the predominant gastric microbiota of healthy humans [20, 21]. The LEFse analysis showed that *Anaerobacillus* were abundant in the GERD environment and could not be found in other diseases. This finding required confirmation by *in-vivo* experiment or meta-transcriptomic and metabolomics analysis to elucidate the potential role of *Anaerobacillus* as a biomarker for ERD. However, it is dissimilar to another study showing that this genus was enriched in superficial gastritis compared to atrophic gastritis and gastric cancer [22]. In another study evaluating the esophageal microbiome during ERD and NERD, there was an increase in Gram-negative bacteria [23]. This study found an enrichment of Gram-negative bacteria such as *Rhizobiaceae, Bacillaceae, Cloacibacterium, Delftia, Desulfovibrio, Pleomorphomonadaceae* and *Tepidomonas*. The enrichment of Gram-negative bacteria may promote the inflammatory signaling through the interaction of bacterial secretomes with the immune system, such as Toll-like Receptors and activate the NF-κB pathway [24]. *Filifactor* and *Stomatobaculum* are bacteria found in mouth plaque and associated with endodontitis and periodontitis. Among 11 enriched bacteria, 5 of them were anaerobic bacteria able to survive in a gastric environment.

*H. pylori* is a bacterium able to dominate gastric microbiota and induce a significant inflammation*.* Nevertheless, the relationship between ERD and *H. pylori* infection remained controversial. Furthermore, our results showed that other factors could also affect ERD, such as smoking habits and alcohol consumption. This result aligns with studies in countries with a high prevalence of *H. pylori* infection [25]. Hence, it is challenging to conclude the relationship between ERD and *H. pylori* which warrants further study.

When all samples were assessed for diversity, the alpha diversity was low in NERD groups, shown by the Simpson and Shannon index, indicating a significant decrease in richness and evenness of microbiota occupying the gastric-antrum environment [26]. The beta diversity analysis also showed a significant separation of the NERD group with many *H. pylori-*positive samples. Moreover, histology and culture results showed that the prevalence of *H. pylori* in the NERD group is higher than in the ERD group. This result showed the strong influence of *H. pylori* infection in NERD through a shift in microbiome composition. The unique finding in the NERD group was consistent with previous study [27]. *H. pylori* infection significantly reduced the alpha diversity as it occupied most of gastric mucosal space [15]. The alpha diversity was low in the NERD group, and beta diversity showed a significant disparity (Fig. 4), while alpha diversity of gastritis and ERD were similar. These results could imply that the presence of *H. pylori* in NERD prevents the development of gastric mucosal erosion, as shown in ERD through its acid regulation [28]. In addition, reflux symptoms observed in NERD patients could overlap with other diseases such as IBD and heartburn related to *H. pylori* infection [29, 30]. Our study results also suggested evaluating *H. pylori* infection in patients with reflux symptoms.

This study has limitations. From patient characteristic, we did not examine other possible differential diagnosis and considered the role of bile reflux in ERD/NERD pathogenesis or other cause of gastritis. GERD subjects without anti-inflammatory cell infiltration either in antrum or corpus was really scarce, hence could potentially inflict a bias in the GERD versus gastritis analysis. We also did not specify the type of PPI usage with a possibility to affect microbial diversity. Moreover, the major limitation of microbiota study through 16S rRNA is the possible contamination, either from other gastrointestinal parts or during sample handling. We attempted to use a sterilized tube and worked under a biosafety cabinet to solve this. Furthermore, ensuring the sequencing quality of each sample is essential. Relatively short sequence length was included to optimize sample number covered in the study. Hence, this may decrease the accuracy of taxonomy classification. Nevertheless, all samples have been rarefied, and the value was standardized.

## Conclusion

The presence of ERD could alter microbiome diversity. A negative correlation between *H. pylori* and microbial diversity in ERD patients is shown in this microbiome study but not in NERD.

## Methods

### Patients and samples

The survey was conducted from 2014 to 2016 in Indonesia to evaluate the correlation between GERD and *H. pylori* infection. Endoscopy and biopsy samples were taken, including 1,074 samples included in our previous study [15]. Biopsy samples were taken in the lesser curvature of the antrum, ~ 3 cm from the pyloric ring. Samples were then stored in a transport medium containing 10% glycerol. We collected demographic data by questionnaire, consisting of age, gender, smoking and alcohol-drinking habit as risk factors for dyspepsia and GERD, as mentioned in our previous study [31]. All subjects aged more than 18 years old, had dyspeptic symptoms (postprandial fullness, early satiety, epigastric pain, and heartburn), and never received *H. pylori* eradication therapy or other antibiotic treatments within 2 weeks prior to examination. We excluded subjects with incomplete specimens and subjects with gastric cancer, duodenal ulcer, gastric ulcer, and severe intestinal metaplasia (gastritis score more than 2). Disease characterization was based on symptoms and upper-endoscopy evaluation. In the beginning, all patients with *H. pylori* positive were classified as gastritis group. The patients presented with reflux symptoms and mucosal lesion (esophageal erosions), which fulfilled the Los Angeles classification were diagnosed with ERD. If the patients presented with reflux symptoms without any mucosal lession (esophageal erosions), NERD was diagnosed. We included subjects without reflux symptoms and no intestinal metaplasia or atrophy score of more than 1 (mild) for histology evaluation in the gastritis group. Randomization was performed to obtain 204 samples for ERD, NERD and gastritis for sequencing. We included only the samples with complete data, especially the questionnaire data. Next, after filtering with a 500 reads cutoff, we finally included 197 subjects for DNA extraction and sequencing (see Additional file 1: Fig. S1).

For microbiome investigation, gastric mucosal specimens from the lesser curvature of the antrum were preserved in a transport medium. For *H. pylori* culture, specimens were placed into 500 µL phosphate-buffered saline, while for DNA extraction we used specimens stored at − 80 °C. Histologic examination was performed with two biopsy specimens from the corpus and antrum to identify the presence of inflammation, atrophy and intestinal metaplasia. All specimens were obtained using Radial Jaw 4 forceps.

All participants signed an informed consent form. The Ethics Committee of Dr. Soetomo Teaching Hospital (Surabaya, Indonesia) and Oita University, Faculty of Medicine (Yufu, Japan) approved the study concept and protocol.

### Determination of H. pylori infection

*H. pylori* obtained from antrum were cultured and incubated at a selective agar plate in microaerophilic conditions for 10 days at 37 °C. Colonies were then sub-cultured in antibiotic-free agar medium; Mueller–Hinton II (Becton Dickinson, Heidelberg, Germany) supplemented with 10% Horse Blood (Nissui Pharmaceutical, Tokyo, Japan), incubated in microaerophilic conditions. *H. pylori* stock was stored at − 80 °C in Brucella Broth (Becton Dickinson, Heidelberg, Germany). All biopsy specimens were fixed in 10% buffered formalin and embedded in paraffin for histologic examination. Hematoxylin eosin, and May-Grunwald Giemsa stain (Beckman-Coulter, California, USA) were used to stain serial sections, and *H. pylori* was detected in samples with bacterial burdens grade 1 or above. Furthermore, we also determined *H. pylori* positive based on the 16S rRNA analysis that reflect colonization with the *H. pylori* abundance cut-off more than 2%, as previously described [15, 32–34].

### DNA extraction and PCR amplification of 16S rRNA sequences

DNA extraction and PCR amplification methods were done as previously described [15]. QIAGEN’s DNeasy Blood & Tissue Kit (QIAGEN, Santa Clarita, California) was used to extract DNA, and DNA Clean & Concentrator was used for concentration (Zymo Research, Irvine, California, USA). Extracted DNA was stored at − 20 °C. Preparation for the 16S rRNA gene library was under the manufacturer’s protocol (Illumina Inc, San Diego, CA). The amplification of V3-V4 of bacterial 16S rRNA gene used universal primers 341F (5′-TCGTCGGCAGCGTCAGATGTGTATAAGAGACAGCCTACGGGNGGCWGCAG-3′) and 805R (5′GTCTCGTGGGCTCGGAGATGTGTATAAGAGACAGGACTACHVGGGTATCTAATCC3-3′).

PCR amplification was performed using KAPA HiFi HotStart Ready Mix (KAPA Biosystem Inc). Eight cycles of PCR were performed using Nextera XT Index kit (Illumina Inc, San Diego, CA). Agencourt AMPure XP magnetic beads (Beckman Coulter, Tokyo, Japan) were used for amplicons purification. Validation of DNA library was achieved with bioanalyzer MCE-202 MultiNA system (Shimadzu, Kyoto, Japan) and QuantiFluor dsDNA system (Promega, Madison, USA). Pooled 5 pM DNA library was denatured with 0.2 N NaOH and combined with PhiX Control v3 (Illumina Inc, San Diego, CA) to 15% of the final concentration, according to Illumina's protocol. MiSeq platform (Illumina Inc, San Diego, CA) and MiSeq Reagent Kit version 3 2 300 bp Paired-End Reads (Illumina Inc, San Diego, CA) were used to perform paired-end sequencing.

### Sequence data analysis

Reads obtained from the Illumina Miseq platform were analyzed in the Qiime2 (Version 2021.2) pipeline [35]. Demultiplexed reads were uploaded and trimmed using cutadapt and filtered to remove low-quality filtering. Denoising, Chimera removal, and clustering were performed by Deblur pipeline that was integrated into the Qiime2. Output was assigned into taxonomy to SILVA 138 reference database with 99% identity parameters [36]. These sequences were aligned using MAFFT, and a phylogenetic tree was constructed using FastTree [37, 38]. We imported the OTU table, representative sequences, and phylogenetic tree as artefacts to Qiime2 analysis platform (https://qiime2.org). The OTU tables, metadata, taxonomy, and phylogenetic tree were exported to phyloseq object using biom-convert and ‘phyloseq’ package in R environment (ver 4.02) for diversity and abundance analysis. The α-diversity analysis, including Observed richness, ACE, Chao1, Fisher, Simpson’s and Shannon's diversity index, were analyzed using ‘vegan’ package in R and produced the figure by ‘ggplot2’ [39]. The β-diversity analysis measured the weighted Unifrac. A Principal component analysis was drawn in R. The Adonis function from the vegan package was used to calculate the permutational analysis of variance (PERMANOVA) with Bonferroni correction. Using the ‘microbiomeMarker’ package in R, we performed linear discriminant analysis effect size (LEfSe) to determine OTUs that are likely to explain differences between GERD, NERD, and gastritis (version 4.02). We also used analysis of variance (ANOVA) to see if there were any differences in the relative abundance of specific OTUs within ethnic groups and diseases.

## Supplementary Information

**Additional file 1.** Supplementary figures.

## Data Availability

The sequences in the dataset are available in the gen bank with accession number PRJNA828345. Available at https://www.ncbi.nlm.nih.gov/sra/PRJNA828345.
