# Supplementary material for: Analysis of gastric microbiota and Helicobacter pylori infection in gastroesophageal reflux disease
Source: Gut Pathog. 2022 Sep 13;14:38. doi: 10.1186/s13099-022-00510-3 (PMC9469549; doi:10.1186/s13099-022-00510-3)
Supplement: Supplementary file 1 — Additional file 1. Supplementary figures. [file 13099_2022_510_MOESM1_ESM.pdf]

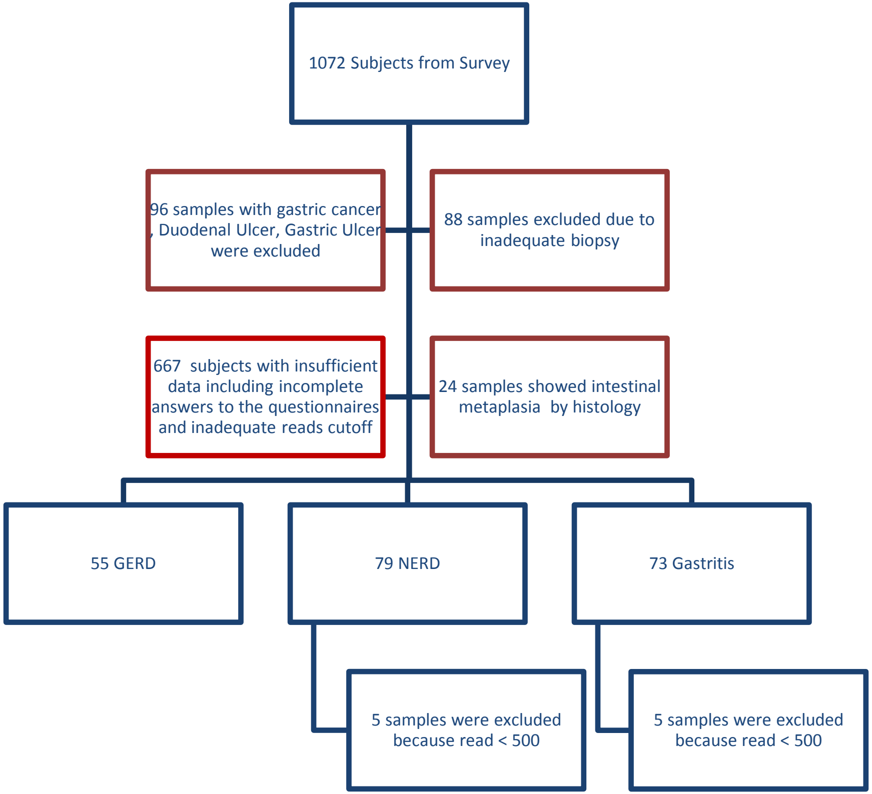

Supplementary Figure S1. Sampling Workflow

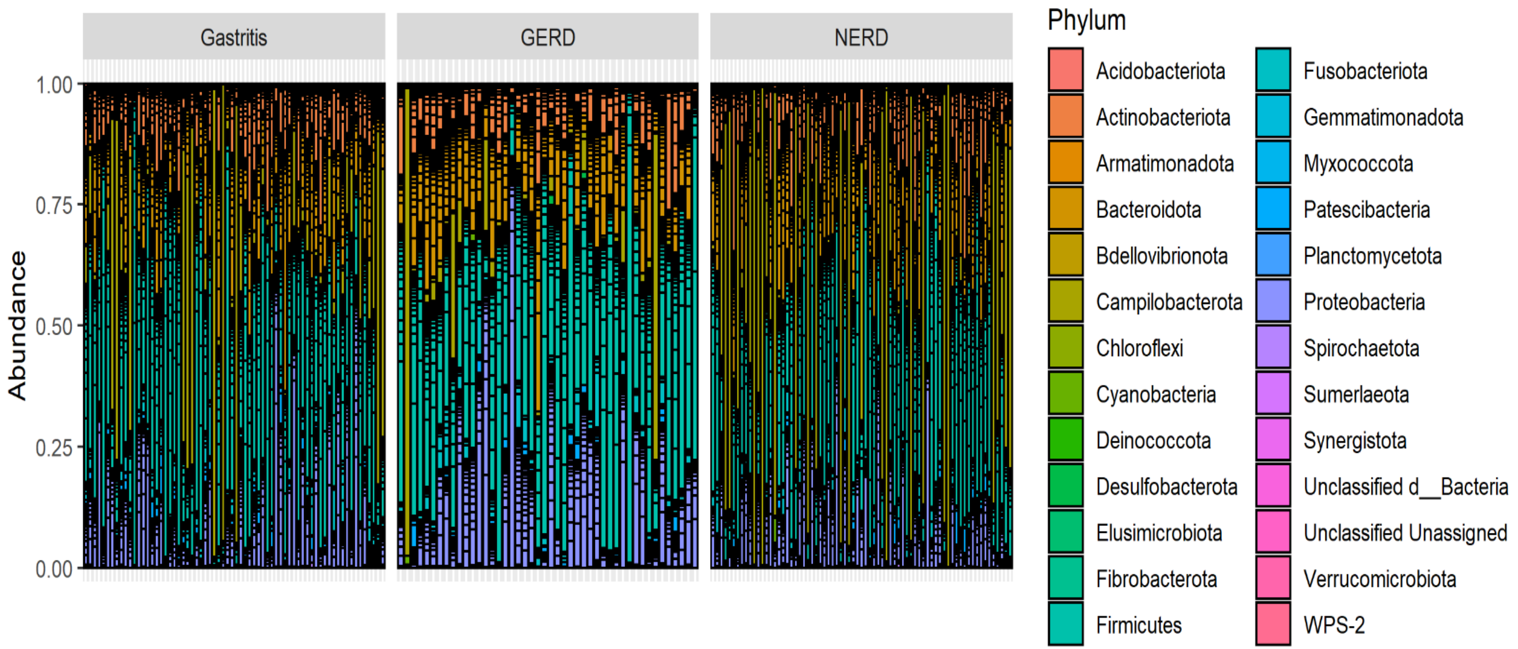

Supplementary Figure S2. The distribution of relative abundance in each sample in the dataset.

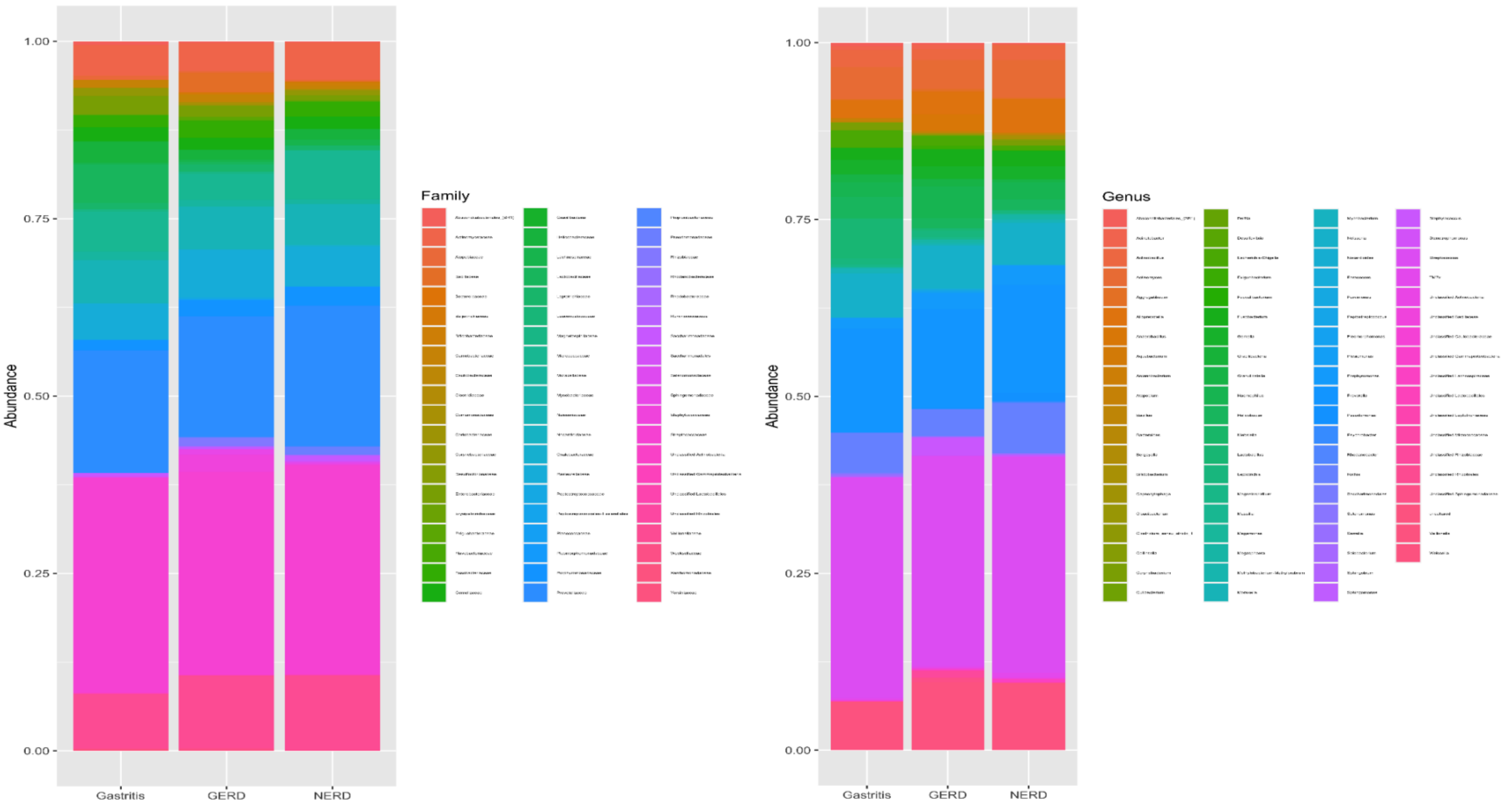

Supplementary Figure S3. Comparison of relative abundance of each diseases group in the Family and Genus.

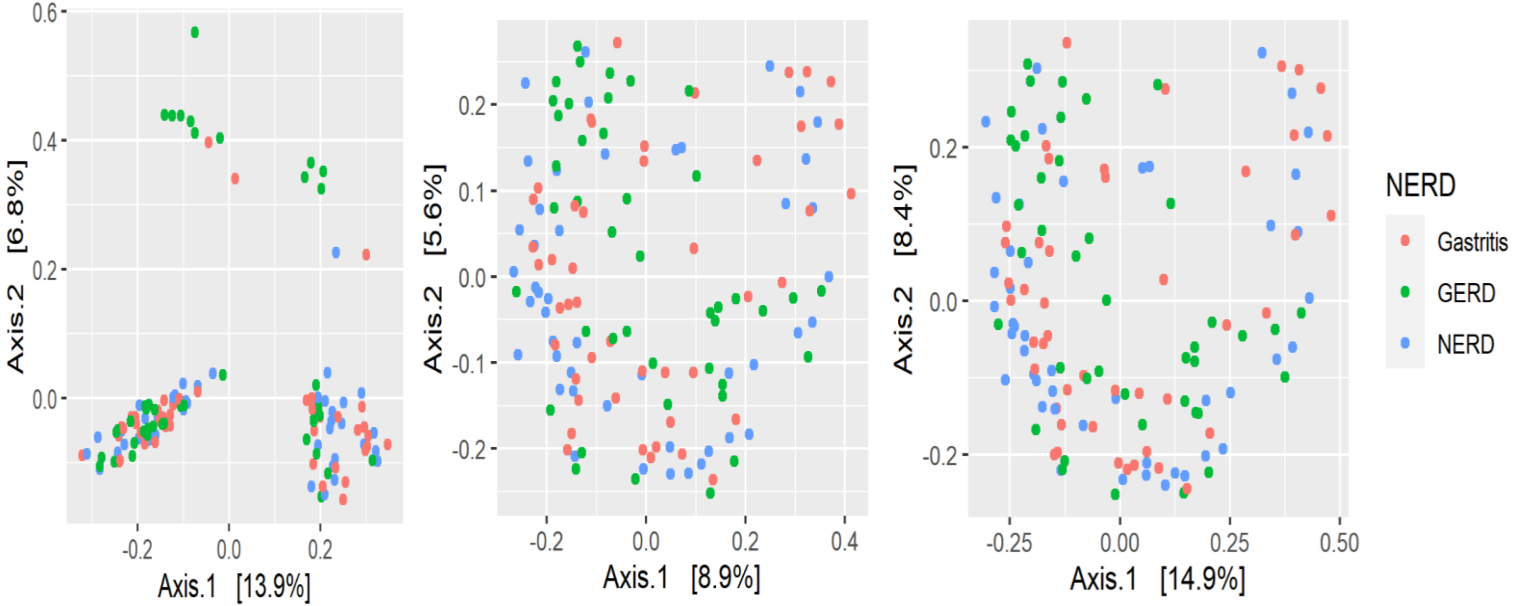

Supplementary Figure S4. The beta diversity measure (from left to right) Unifrac, Bray-Curtis, and Jaccard between gastritis, GERD, and nerd group after excluding the samples with *H. pylori.*

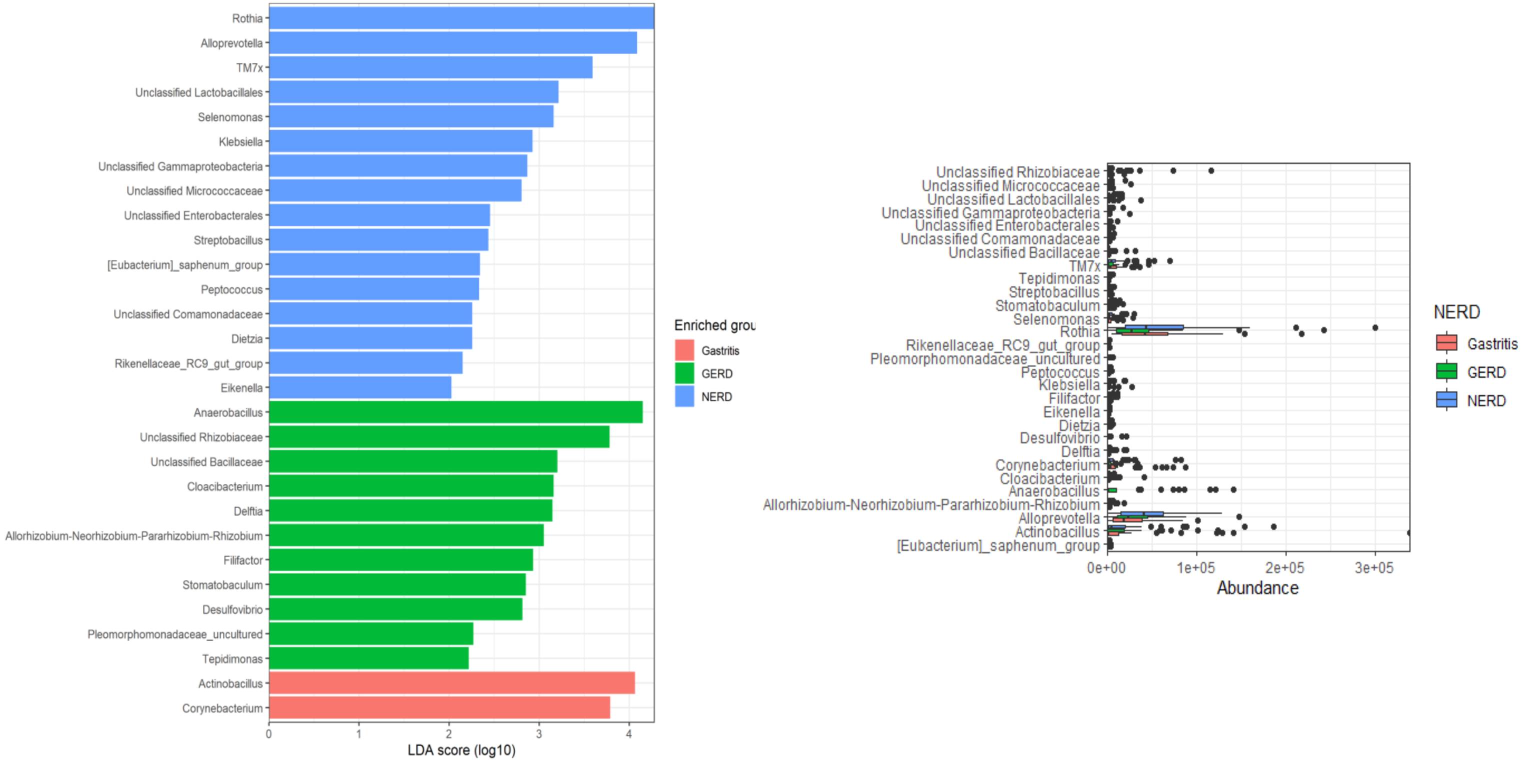

Supplementary Figure S5 Analysis of LDA by LEFse of the genus level found possible biomarker for GERD, NERD and Gastritis (Upper). The relative abundance of each genus was represented as boxplot.

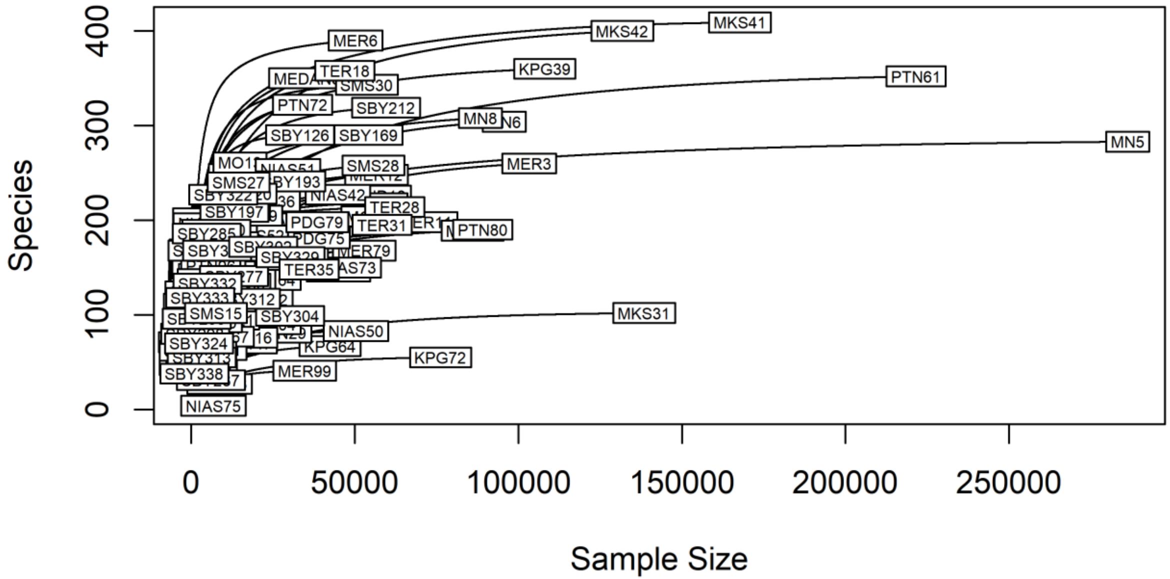

Supplementary Figure S6. Rarecurve figure of the samples in dataset

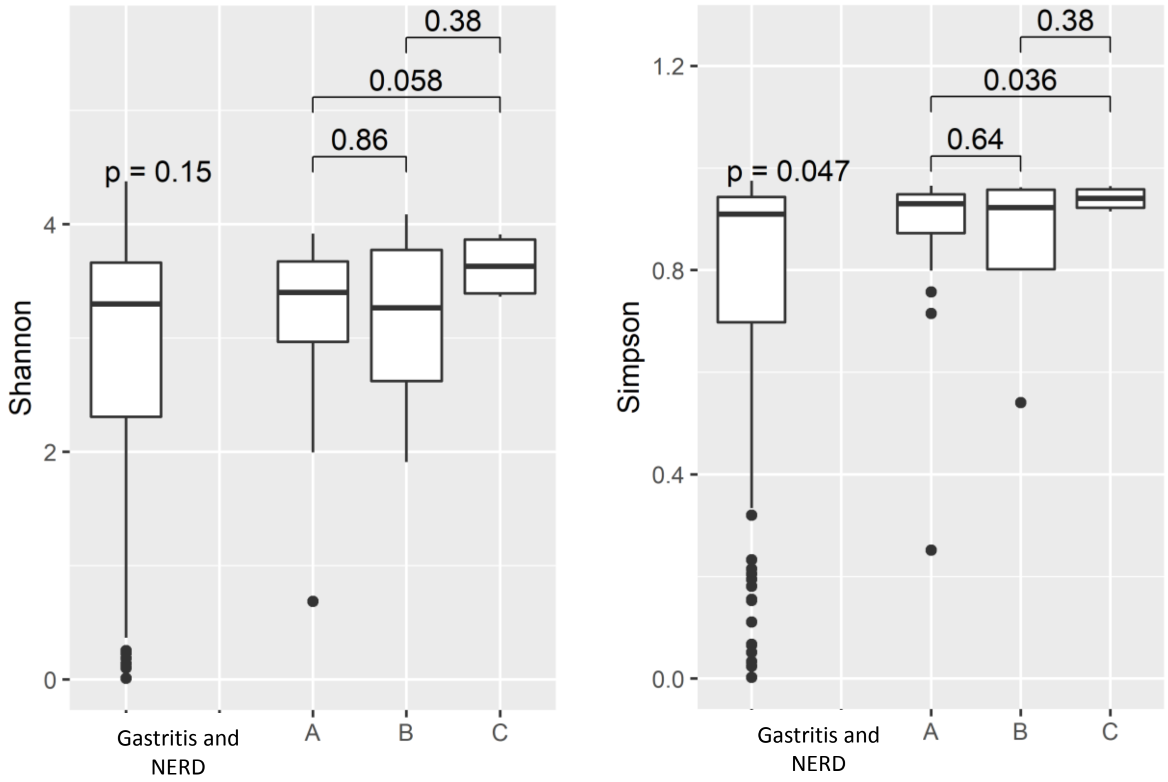

Supplementary Figure S7. Comparison among ERD severity. The groups in the comparison were Gastritis-NERD, LA-A, LA-B and LA-C
